# Rapid Nanophotonics Assay for Head and Neck Cancer Diagnosis

**DOI:** 10.1038/s41598-018-29428-0

**Published:** 2018-07-30

**Authors:** P. Vohra, P. Strobbia, H. T. Ngo, W. T. Lee, T. Vo-Dinh

**Affiliations:** 1 Department of Biomedical Engineering, Duke University, Durham, NC USA; 2 Department of Chemistry, Duke University, Durham, NC USA; 3 Fitzpatrick Institute for Photonics, Duke University, Durham, NC USA; 4 Division of Head and Neck Surgery and Communication Sciences, Duke School of Medicine, Durham, NC USA; 5 Present Address: Biomedical Engineering Department, International University, Vietnam National University-Ho Chi Minh City (VNU-HCMC), Ho Chi Minh City, Vietnam

## Abstract

Efficient and timely diagnosis of head and neck squamous cell carcinoma (HNSCC) is a critical challenge, particularly in low and middle income countries. These regions, which are expected to witness a drastic increase in HNSCC rates, are ill-prepared to handle the diagnostic burden due to limited resources, especially the low ratio of pathologists per population, resulting in delayed diagnosis and treatment. Here, we demonstrate the potential of an alternative diagnostic method as a low-cost, resource-efficient alternative to histopathological analysis. Our novel technology employs unique surface-enhanced Raman scattering (SERS) “nanorattles” targeting cytokeratin nucleic acid biomarkers specific for HNSCC. In this first study using SERS diagnostics for head and neck cancers, we tested the diagnostic accuracy of our assay using patient tissue samples. In a blinded trial, our technique demonstrated a sensitivity of 100% and specificity of 89%, supporting its use as a useful alternative to histopathological diagnosis. The implications of our method are vast and significant in the setting of global health. Our method can provide a rapid diagnosis, allowing for earlier treatment before the onset of distant metastases. In comparison to histopathology, which can take several months in remote limited-resources regions, our method provides a diagnosis within a few hours.

## Introduction

Affecting over 600,000 people worldwide each year^1^, head and neck cancers present a major issue in the field of global health. These tumors can significantly impact basic life functions such as swallowing, breathing, and speaking. Head and neck squamous cell carcinomas (HNSCCs), which comprise over 90% of these tumors^2^, can be curable if diagnosed at an early stage. Despite advances in diagnostic and therapeutic technology, HNSCCs continue to comprise a large portion of the global cancer burden. This is largely due to an epidemiological shift towards low and middle income countries (LMICs). While rates of head and neck squamous cell carcinomas are declining in the United States, their incidence is on the rise in LMICs countries^3^ due to risk factors including smoking, drinking, betel nut chewing, and HPV infection^4,5^. Two thirds of HNSCCs now occur in developing countries^6^. By 2030, the projected incidence of HNSCC will exceed 1 million, 73% of which will arise from less developed countries^6,7^.

The rise of HNSCC in LMICs is alarming and poses a major challenge to global health. Diagnostic capacity in these regions is limited and inadequate to handle the increased burden. In developed countries, HNSCC is traditionally diagnosed using the gold standard of histopathological analysis and immunohistochemical staining for cytokeratin. However, in much of the world, a single pathologist often serves a population of several million people^8,9^. Diagnosis of HNSCC can take weeks or months, thereby delaying treatment. As the incidence of HNSCC rises, particularly in LMICs, there is a growing need for alternative diagnostic approaches.

A common presentation of HNSCC is an enlarged cervical lymph node. However, lymphadenopathy is not specific for HNSCC and may be attributed to a multitude of diagnoses including thyroid carcinoma, viral infection, tuberculosis, and lymphoma, among others. In regions with limited access to pathologists, this poses a barrier to treatment for patients suffering from HNSCC. Our group sought to develop a resource-efficient method to distinguish the presence of HNSCC from other pathologies in human lymph nodes, permitting earlier diagnosis and treatment before widespread metastasis, thereby improving survival.

**Figure 1:**
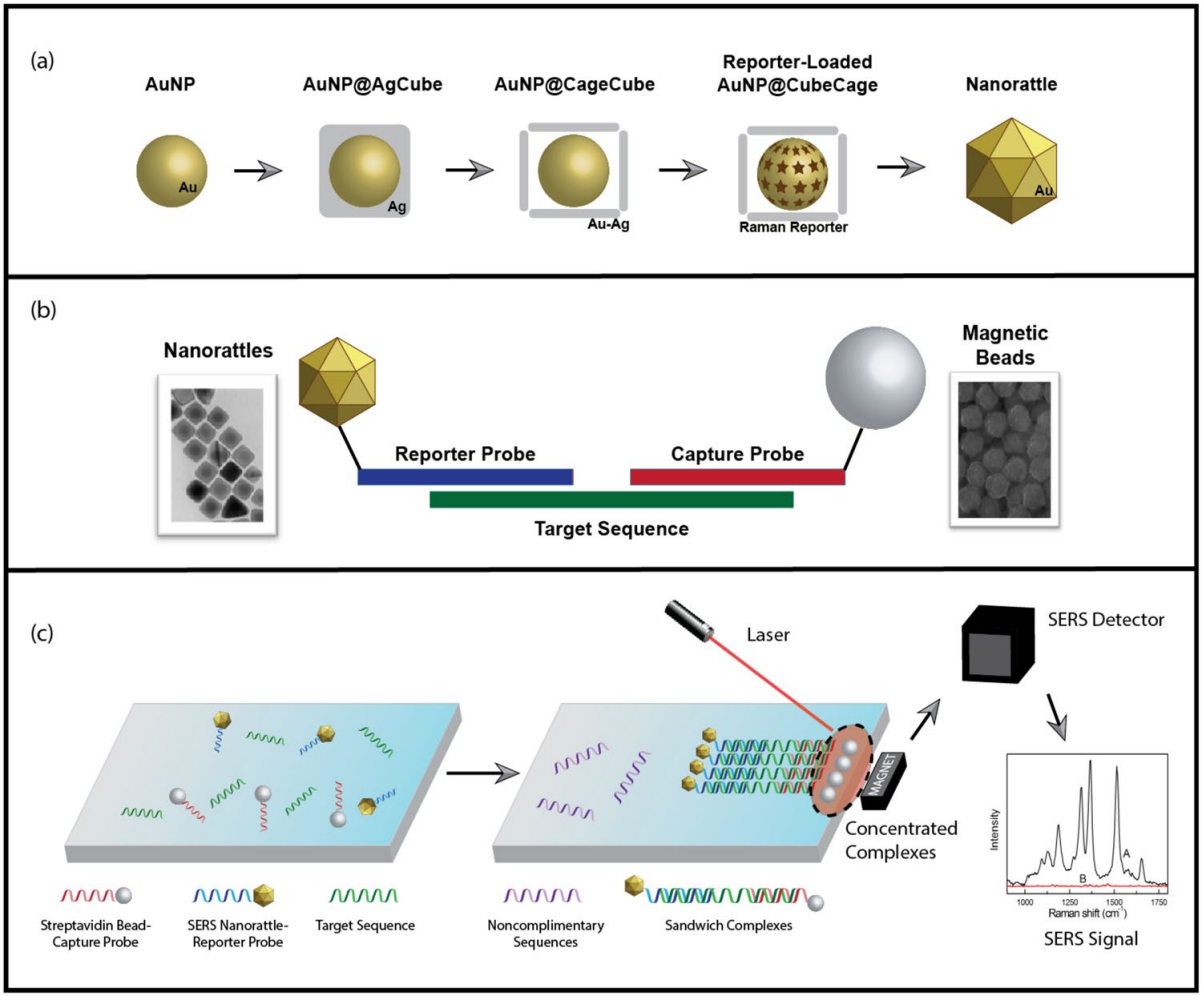
Overview of SERS Diagnostic Method. (**a**) Synthesis of cubic nanorattles, beginning with single-crystal, spherical gold nanoparticle (AuNP) cores. The AuNP cores are coated with cubic Ag shells to obtain AuNP@AgCube. Galvanic replacement transforms cubic Ag shells into cubic Au-Ag cages containing AuNP in the interior. Raman reporters are loaded, and the porous cubic cages are turned into complete shells by a final Au coating to obtain cube nanorattles. (**b**) Structure of individual hybridization complex. Gold nanorattles (Au-NR) are functionalized with DNA reporter probes, and streptavidin beads are functionalized with DNA capture probes. Both probes are complimentary to the specific cytokeratin sequence. (**c**) Extracted nucleic acids are incubated with functionalized nanorattles and beads, then washed away. Remaining complexes undergoing successful hybridization are isolated using a strong magnet, and concentrate to a spot for laser excitation in order to yield a SERS signal.

Raman spectroscopy offers an approach to molecular diagnosis by providing unique chemical fingerprints produced from the inelastic scattering of light upon interacting with distinct molecules. Surface-enhanced Raman scattering (SERS) further enhances the signals by providing magnification on the order of millions, allowing for single molecule detection^10–12^. SERS has been developed in our laboratory for many applications including chemical monitoring, biosensing and molecular diagnostics^13–17^. Recently we have developed a sensitive SERS sandwich hybridization assay using unique SERS nanorattles for nucleic acid detection^18^. Unlike previous applications of sandwich hybridization, which utilized gold and silver nanoparticles, the unique core-gap-shell structure of our nanorattles results in ultrabright SERS signals, and thus higher sensitivity for nucleic acid detection^19,20^. This method is summarized in Fig. 1. Firstly, SERS nanorattles and magnetic beads are hybridized to unique nucleic acid sequences. Following magnetic isolation, a laser is applied to the hybridized complexes. When successful hybridization occurs in the presence of target nucleic acid sequences, an increase in SERS signal can be detected.

We have previously described a surface-enhanced SERA sandwich assay utilizing unique nanoparticles to detect cytokeratin nucleic acid biomarkers in HNSCC^21^. Cytokeratin 14 (CK14) mRNA has been demonstrated to be both sensitive and specific for detection of HNSCC micrometastases in lymph nodes^22^. In contrast to other members of the cytokeratin family of proteins, CK14 is specific for head and neck SCC, and can distinguish SCC from thyroid tumors^23–25^. In the present study, we employ our diagnostic method in human tissue samples. This study is the first of its kind to use SERS nanoparticles to diagnose HNSCC *ex vivo* using clinical specimens. Using our novel sandwich hybridization protocol, we demonstrate the diagnostic capacity of our method in a blinded trial of 25 patient samples.

## Results

A total of 25 samples were obtained from human cervical lymph nodes, tonsils, oropharyngeal mucosa, sinus mucosa, and thyroid gland tissue. Histopathological diagnoses ranged from HNSCC, normal lymphoid tissue, follicular lymphoid hyperplasia, normal oral mucosa, normal thyroid tissue, papillary thyroid carcinoma, and melanoma. A summary of tissue sample characteristics is shown in Table 1.

**Table 1:** Tissues and diagnoses of clinical samples.

Sample	Tissue Site	Histopathological Diagnosis
1	thyroid	papillary carcinoma
2	thyroid	normal thyroid tissue
3	lymph node	squamous cell carcinoma
4	oral mucosa	squamous cell carcinoma
5	oral mucosa	squamous cell carcinoma
6	tongue	normal squamous mucosa
7	lymph node	papillary carcinoma
8	thyroid	papillary carcinoma
9	thyroid	normal thyroid tissue
10	lymph node	squamous cell carcinoma
11	tongue	normal squamous mucosa
12	lymph node	papillary carcinoma
13	sinus	squamous cell carcinoma
14	tongue	squamous cell carcinoma
15	lymph node	papillary carcinoma
16	tongue	squamous cell carcinoma
17	lymph node	follicular lymphoid hyperplasia
18	tonsil	normal lymphoid tissue
19	tonsil	normal lymphoid tissue
20	lymph node	melanoma
21	tonsil	normal lymphoid tissue
22	tonsil	normal lymphoid tissue
23	lymph node	melanoma
24	tonsil	normal lymphoid tissue
25	tonsil	normal lymphoid tissue

**Figure 2:**
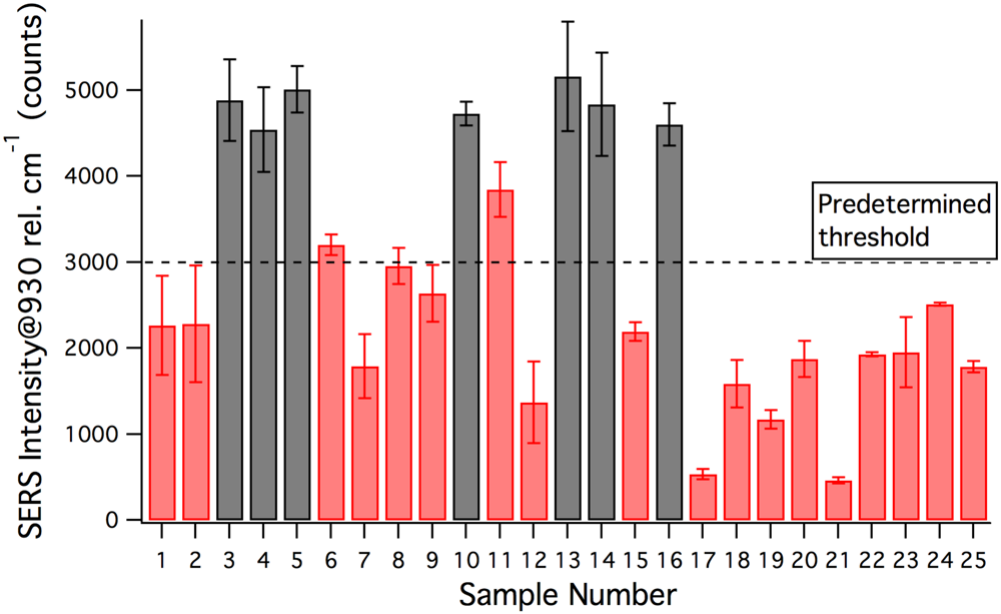
Blinded Test: Intensities of 930 cm^−1^ peaks against threshold.

Using our cytokeratin detection probes, our method was applied to each tissue sample in triplicate. The SERS intensities were quantified by the peak intensity at 930 cm^−1^. In our previous study using human cell lines, we derived a threshold of 3000 counts to distinguish HNSCC from other cell types. We applied the same threshold to our tissue samples, as depicted in Fig. 2. Using our method, we diagnosed samples above this threshold as HNSCC and those below as negative for HNSCC.

Our diagnostic impressions were then compared to the histopathological diagnosis of each tissue sample. The sensitivity and specificity of our method was calculated, as shown in Table 2.

**Table 2:** Sensitivity and specificity of SCC detection in blinded samples.

	SCC	Non-SCC	Total
Total	7	18	25
Test Positive	7	2	9
Test Negative	0	16	16
	**Sensitivity** 7/7 = **100%**	**Specificity** 16/18 = **89%**	

**Figure 3:**
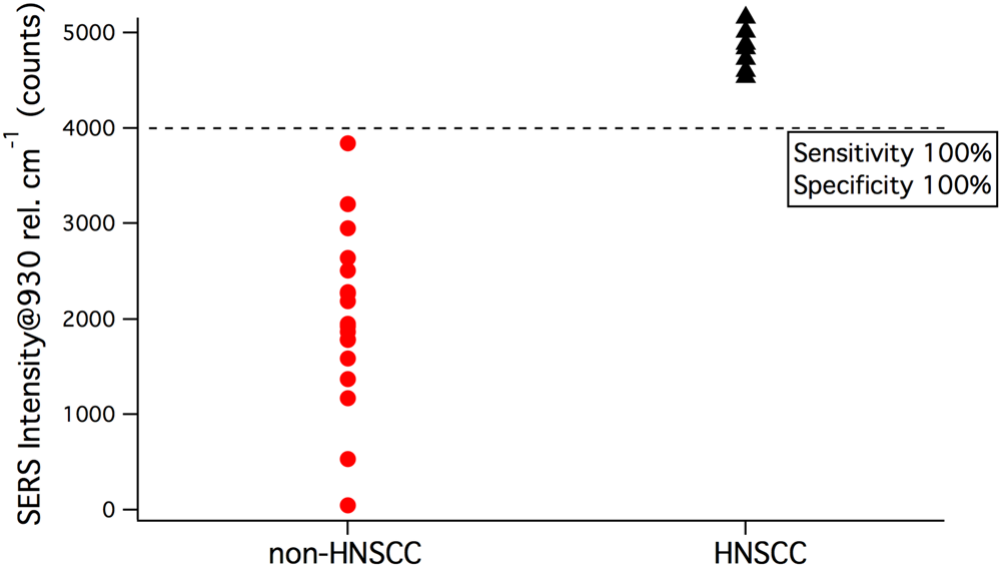
Distribution of SERS peaks by diagnosis after unblinding.

Following the calculation of sensitivity and specificity, the distribution of samples, now un-blinded, was analyzed. Figure 3 depicts the distribution of non-HNSCC compared to HNSCC. A threshold of 4000 counts was observed to demarcate the two groups with 100% sensitivity and 100% specificity.

## Discussion

SERS-based methods are becoming increasingly popular in nucleic acid detection due to their high sensitivity. We previously developed a SERS method to detect HNSCC as a diagnostic alternative to histopathology using human cell lines^21^. In the present study, we validated our unique SERS method in human tissue samples. By targeting cytokeratin 14 RNA with ultrabright SERS nanorattles, we demonstrated the capability of our method in distinguishing HNSCC from other tissue types such as thyroid cancer and benign lymphoid tissue. In a blinded trial of 25 samples, our method demonstrated 100% sensitivity and 89% specificity for HNSCC detection, supporting its use as an alternative to histopathological diagnosis.

In our blinded test, we used a previously derived threshold of 3000 counts to diagnose HNSCC. Using this threshold value, our method was able to correctly diagnose all 7 HNSCC samples. Out of the remaining 18 negative samples, 16 were correctly diagnosed as non-HNSCC. The 2 negative samples that tested positive were both squamous tissue from the oral mucosa. While the signals from these tissues exceed the threshold, the data demonstrate that these samples exhibit lower SERS signals than HNSCC. Furthermore, upon unblinding, we identified a new threshold of 4000 counts to delineate HNSCC from non-HNSCC. Compared to the previous threshold, which was derived using data from human cell lines, the new threshold resulted in 100% sensitivity and 100% specificity. As this was derived from human clinical samples, it represents a significant improvement to our previous method, and will be further evaluated in future studies using larger sample sets.

This is the first study using SERS diagnostics for head and neck cancers in clinical specimens. The use of solid tissue samples demonstrates the capacity of our method to detect nucleic acid sequences within the complex, structural framework of the tissues. However, for practical application of our method in the clinical setting, fine needle aspirate (FNA) samples will be required as they are less invasive. In future studies, we will optimize our method for detection in fine needle aspirate (FNA) samples. We anticipate that due to the relative purity of cells in FNA samples compared to solid tissue, and previous work supporting molecular testing in FNA samples^26^, our method will be effective.

The results of our study serve to establish the use of SERS detection of HNSCC in human tissue samples. We anticipate that ultimately this method can be incorporated into an integrated device that can be used in the field for point-of-care testing as a diagnostic alternative to histopathology. Our team recently developed a prototype of an integrated diagnostic device capable of detecting malaria in human blood lysate^27^. For patients presenting with cervical lymphadenopathy, our method can provide a diagnosis in a more timely manner than the current standard, thereby allowing for earlier treatment.

The implications of our method are vast and significant in the setting of global health. For patients presenting cervical lymphadenopathy, our method can provide a rapid diagnosis, allowing for earlier treatment before the onset of metastases. Compared to diagnosis by histopathology, which can take several months in remote regions, our method can be completed in a matter of a few hours. While digital pathology services have emerged to meet the growing diagnostic demand, these telemedicine technologies face many challenges, including limited infrastructure, incompatibility among different platforms, security, and resistance to cultural change^28,29^. Our method can be completed by a local technician and provide patients with a point-of-care diagnosis without transferring personal health data.

Additionally, our method has the advantage of incorporating molecular testing. For head and neck cancers, this is of particular importance as HPV status is an important prognosticator^30^. Furthermore, our method does not require the expensive equipment necessary for qRT-PCR; a simple thermal cycler powered by solar energy is adequate and affordable^31^. Finally, as our method yields an output of a binary diagnosis based on SERS signal intensity, rather than a qPCR curve, it can be easily interpreted in the field. Our method thus provides a cheaper, user-friendly, resource-efficient alternative for use in LMICs.

## Methods

### Patient Selection

For this prospective study, eligible patients included adults with HNSCC, thyroid papillary carcinoma, lymphoma, or benign lymphoid or tonsillar disease. Patients with cutaneous squamous cell carcinoma were excluded from the study. Patients were identified on the basis of undergoing head and neck surgery at Duke University in 2017. The study was approved by the Duke institutional review board. All experiments were performed in compliance with the relevant guidelines and regulations. Informed consent was obtained from all patients by the Duke Biospecimen Repository and Processing Core (BRPC). Participation in the study did not alter the management of patients.

### Collection of Clinical samples

Fresh tissue samples were collected intraoperatively by the primary surgeon. After adequate tissue was dissected by Duke Pathology for standard histopathological diagnosis, remaining tissue was collected by the Duke BRPC. Samples were flash frozen in liquid nitrogen and maintained at −80 °C. Our team was blinded to all diagnostic information and received de-identified tissues containing only a unique numerical identifier.

### RNA Extraction and Amplification

All steps involving fresh tissues and RNA were conducted on ice. 30 mg of tissue was required for RNA extraction. Tissues were first homogenized using a mortar and pestle and subsequently with a 21 gauge needle and syringe. RNA extraction was performed on all tissue samples using the Qiagen RNAeasy Mini Kit. RNA was eluted in RNAse free deionized water (dH2O) to final concentrations between 100–300 ng/uL. RNA was normalized to a concentration of 100 ng/uL. then immediately used for amplification as per the Qiagen OneStep PCR Kit with our previously designed primers and probes^21,22^. Briefly, 5uL of RNA was added to a mixture containing 1000 nM forward primer, 50 nM reverse primer, 200 nM fluorescent probe, 2uL RT-PCR Buffer 5X, 2uL OneStep RT-PCR Enzyme Mix, and 30uL RNase-free DI to a final volume of 50 mL. Samples were heated for 5 minutes at 95 C, then amplified by heating to 60 C for 1 minute. This was followed by 40 cycles of 15 s at 95 C. The Qiagen PCR Purifcation Kit was used to remove any excess products, and the final single stranded cDNA was eluted in RNase-free DI to final volumes of 7–18 ng/mL.

### Nanorattles and Magnetic Beads Preparation

#### Materials

Sodium borohydride (NaBH4), Au chloride solution (HAuCl4) 200 mg/dL in deionized water, ascorbic acid (AA), hexadecyltrimethylammonium chloride (CTAC) solution 25 wt% in water, polyvinylpyrrolidone Mw ~55,000 (PVP), ethanol (EtOH), 1,3,3,1′,3′,3′,-hexamethyl-2,2′-indotricarbocyanine iodide (HITC), phosphate buffer saline (PBS), tris-EDTA buffer solution (TE), tween 20, sodium chloride (NaCl), hydrochloric acid (HCl) were purchased from Sigma-Aldrich. Sodium citrate dihydrate was purchased from BDH. Methoxy polyethylene glycol thiol Mw 5000 (mPEG-SH) was purchased from Nanocs. Magnetic beads were purchased from Life Technologies. All DNA sequences were synthesized by Integrated DNA Technologies (IDT, Coralville, IA). Millipore Synergy ultrapure water (DI) of resistivity = 18.2 MΩ cm was used in all nanoparticle synthesis solutions. Nuclease-free water was used in all experiments relating to RNA and DNA.

#### Nanorattles Synthesis

Nanorattles were synthesized with a previously developed procedure^18^. In brief, the gold nanoparticles (GNPs) were prepared using a seed-mediated method, resulting in 20 nm GNPs^32^. This method consisted in the synthesis of gold seeds by the reduction of gold chloride by NaBH4 in ice bath and the further growth of the seeds obtained by reduction of gold chloride by ascorbic acid the presence of CTAC. After washing the GNPs suspension, a silver shell was coated on the GNPs by reducing AgNO3 with ascorbic acid in the presence of CTAC at 60 °C for 4 h. This reaction yields gold-silver core-shell structures with a cubic shape (GNP@AgCubes). The silver shells were then converted into cages using galvanic replacement. A solution of Au chloride was slowly added to the GNP@AgCubes in the presence of PVP and CTAC at 90 °C, obtaining the porous mixed gold-silver shells. For Raman reporter loading, the stock GNP@AgCages were mixed with 1 mM HITC in ethanol. After 2 h under shaking, the suspension was washed for the final gold coating, which was performed by reducing gold chloride with ascorbic acid in the presence of CTAC.

#### Nanorattles Functionalization

Nanorattles were functionalized with DNA reporter probes using a pH-assisted method with slight modification^18^. First, 5 µL of 100 mM TCEP in TE 1X were added to 50 μL of 100 μM thiolated DNA reporter probes. The mixture was added to 1 mL of nanorattles after 1 h incubation at rom temperature. This new mixture was incubated for 1 h under shaking. Then 10 μL of citrate-HCl buffer (300 mM trisodium citrate, pH adjusted to 3.1 using 1 M HCl) were added to promote loading of DNA onto nanorattles and incubated for 1 more hour. The mixture was centrifuged at 6,500 rpm for 5 minutes. Fifty µL of 1 mM SH-PEG 5 K (freshly prepared, sonicated 5 minutes) were added to the pellet followed by addition of 1 mL PBS 1X with 0.01% tween20 and sonication. The resulting nanorattles were washed once with TE 1X followed by resuspension in TE 1X and storage at 4 °C before use. The functionalized nanorattles are good for use within 3–4 months.

#### Magnetic Beads Functionalization

Magnetic beads (Dynabeads MyOne Streptavidin C1, 1 μm diameter) were functionalized with DNA capture probes using the manufacturer’s protocol. Briefly, 800 µL of10 mg/mL stock magnetic beads were washed three times using washing buffer 1X (5 mM Tris-HCl pH 7.5, 0.5 mM EDTA, 1 M NaCl) and suspended in 400 µL of washing buffer 2X. To load DNA capture probes on magnetic beads, 400 μL of 5 μM biotinylated DNA capture probe were added. The mixture was incubated for 0.5 h under shaking. DNA capture probe-loaded magnetic beads were washed three times using washing buffer 1X and resuspended in 1600 μL TE 1X (magnetic bead final conc. 1.25 mg/ml). The capture probe-loaded magnetic beads solution was stored at 4 °C and used within 3–4 months.

### DNA Detection

Target sequence detection capability of the method was demonstrated by testing sample solutions containing purified PCR products of extracted RNA in hybridization buffer (0.5 M NaCl, 10 mM phosphate-buffered solution at pH 7.4) with Tween-20 0.01%. For each sample, 3 µL DNA, 2 μL of magnetic beads loaded with capture probes, and 3 μL of nanorattles functionalized with reporter probes were added to 27 µL of buffer. The samples were incubated at 40 C for 3 hours, then washed three times before being pipetted into glass capillary tubes (5–25 μL volume, Sigma-Aldrich). The magnetic bead complexes were concentrated at small spots near the middle of the capillary tubes using a permanent magnet positioned under the tubes.

### SERS Measurement

Using a lab-built system, a laser beam was focused onto the concentrated magnetic bead complexes. The lab-built SERS measurement system was composed of a 785 nm laser source (Rigaku Xantus-1), a fiber optic probe (InPhotonics RamanProbe), a spectrometer (Princeton Instruments Acton LS 785), and a CCD camera (Princeton Instruments PIXIS: 100BR_eXcelon). Laser power of the Xantus-1 was set at 300 mW and the CCD camera exposure time was set at 1 second.
