# Author Correction: Rapid Nanophotonics Assay for Head and Neck Cancer Diagnosis

**DOI:** 10.1038/s41598-018-31277-w

**Published:** 2018-08-23

**Authors:** P. Vohra, P. Strobbia, H. T. Ngo, W. T. Lee, T. Vo-Dinh

**Affiliations:** 1 Department of Biomedical Engineering, Duke University, Durham, NC USA; 2 Department of Chemistry, Duke University, Durham, NC USA; 3 Fitzpatrick Institute for Photonics, Duke University, Durham, NC USA; 4 Division of Head and Neck Surgery and Communication Sciences, Duke School of Medicine, Durham, NC USA; 5 Present Address: Biomedical Engineering Department, International University, Vietnam National University-Ho Chi Minh City (VNU-HCMC), Ho Chi Minh City, Vietnam

Correction to: *Scientific Reports* 10.1038/s41598-018-29428-0, published online 30 July 2018

The original version of this Article contained a typographical error in the spelling of the author T. Vo-Dinh, which was incorrectly given as T. Vo Dinh. This has now been corrected in the PDF and HTML versions of the Article.

